# Letter from Prof. J. G. Thomas, of Savannah, Ga.

**Published:** 1871-04

**Authors:** J. G. Thomas

**Affiliations:** Savannah, Ga.


### PART III.

### Miscellaneous Correspondence.

### LETTER FROM PROF. J. G. THOMAS, M. D., SAVANNAH, GA.

SAVANNAH, GA., March 16, 1871.

*Drs. Powell & Goldsmith.—Gentlemen :*—I have noted a little matter this winter, in my teachings of auscultation and percussion, and desire to throw it out like a waif to the profession.

This allusion is to the influence of the state of the atmosphere upon the sounds heard by auscultation, and, more particularly, in percussion. There is no one, I suppose, but has realized that there were times when they would be able to make out their cases by these physical means much more clearly than at others. Take, for example, a case where there is but a small arear of the apex of the lung affected by tuberculosis, producing very slight dulness, and increasing the *pitch* of the sound produced by percussion. We all know there are times when the ear will detect that increase of pitch or slight dulness much more readily, and we feel much more certain of its existence than at others. Again, in cases where we have very little hypertrophy of the heart, and the arear of dulness very little increased, and we desire to make out the precise line where dulness ceases, and thus define the outlines of the heart, we find we accomplish this easier and are much more sure of our lines being correct at one time than at another. For a long time I attributed all this to differences in attention, perception, and perhaps little more acuteness of hearing at one time than at others. But, as intimated above, last winter, whilst instructing my class in the Savannah Medical College, I became satisfied that the state of the atmosphere had more or less to do with it. There is no farmer who has a dinner horn but has often realized that the atmosphere is a much better conductor of sounds at times than at others, and that this is not altogether due to stillness of this medium; but is doubtless owing to the amount of moisture, difference in rarification, etc. But what I desire is to call the attention of the profession to these facts, and to see if the idea is verified by others.

Shortly after becoming convinced of the truth of these things, I wrote to Prof. Austin Flint, of New York, upon the point, and his reply was in these words: "Your enquiry contains an idea which has never occurred to me. As I have never thought of it before, I of course can say nothing respecting it but to "guess" that there is more or less truth in it. In appreciating slight differences in sounds, elicited by percussion and heard in ausculation, I know there is much difference as regards myself at different times; but I have attributed this to variations as regards mental attention incident to dullness, preoccupation, etc."

Dr. F. goes on to say that "there is one objection to bringing forward the idea," and that is that it would "tend to overestimate the amount of delicacy in such things"; and that he has been accustomed, in order to encourage this branch of practical medicine, to teach that no great amount of delicacy of perception was necessarary for all ordinary purposes. Now, whilst I can fully appreciate his proposition, and can say that I endeavor to make the same impressions upon my students, yet must differ as to the policy of bringing it forward. I feel that everything that can be observed in connection with this valuable mode of diagnosis should be noted and given to the profession at large. For this reason I have trespassed upon your valuable space, hoping that it may call out other opinions in the same direction.

Wishing you every success in your new enterprise, I remain yours, respectfully,

J. G. THOMAS, M. D.

